# The Anti-Inflammatory Effect of Spray-Dried Plasma Is Mediated by a Reduction in Mucosal Lymphocyte Activation and Infiltration in a Mouse Model of Intestinal Inflammation

**DOI:** 10.3390/nu8100657

**Published:** 2016-10-22

**Authors:** Anna Pérez-Bosque, Lluïsa Miró, Concepció Amat, Javier Polo, Miquel Moretó

**Affiliations:** 1 Departament de Bioquímica i Fisiologia, Facultat de Farmàcia i Ciències de l’Alimentació and Institut de Nutrició i Seguretat Alimentària, Universitat de Barcelona (UB), 08028 Barcelona, Spain; lluisa.miro@ub.edu (L.M.); camat@ub.edu (C.A.); mmoreto@ub.edu (M.M.); 2 APC-Europe S.A., 08403 Granollers, Spain; javier.polo@apc-europe.com

**Keywords:** intestinal inflammation, *S. aureus* enterotoxin B, adhesion molecules, transcription factors, dietary supplementation

## Abstract

Spray-dried preparations from porcine and bovine plasma can alleviate mucosal inflammation in experimental models and improve symptoms in patients with enteropathy. In rodents, dietary supplementation with porcine spray-dried plasma (SDP) attenuates intestinal inflammation and improves the epithelial barrier function during intestinal inflammation induced by *Staphylococcus aureus* enterotoxin B (SEB). The aim of this study was to discern the molecular mechanisms involved in the anti-inflammatory effects of SDP. Male C57BL/6 mice were fed with 8% SDP or control diet (based on milk proteins) for two weeks, from weaning until day 33. On day 32, the mice were given a SEB dose (i.p., 25 µg/mouse) or vehicle. SEB administration increased cell recruitment to mesenteric lymph nodes and the percentage of activated Th lymphocytes and SDP prevented these effects). SDP supplementation increased the expression of interleukin 10 (IL-10) or transforming growth factor- β (TGF-β) compared to the SEB group. The SEB challenge increased six-fold the expression of mucosal addressin cell adhesion molecule 1 (MAdCAM-1) and intercellular adhesion molecule 1 (ICAM-1); and these effects were attenuated by SDP supplementation. SEB also augmented NF-κB phosphorylation, an effect that was prevented by dietary SDP. Our results indicate that the anti-inflammatory effects of SDP involve the regulation of transcription factors and adhesion molecules that reduce intestinal cell infiltration and the degree of the inflammatory response.

## 1. Introduction

Stress, xenobiotics, food components, environmental toxins or changes in the microbiota may trigger intestinal inflammation, which involves increased production of pro-inflammatory cytokines. This inflammatory syndrome may be worsened by the genetic susceptibility of exposed individuals, as observed in patients suffering inflammatory bowel diseases [1]. The inflammatory response is initiated by the mucosal gut-associated lymphoid tissue (GALT), which is responsible for local responses as well as for phenomena that occur in other mucosal regions, such as nasal-associated lymphoid and genitourinary-associated lymphoid tissues [2].

It is well documented that colostrum and breast milk are a source of immunoglobulins and other proteins that contribute to the early development of the immune system and that they have protective tolerogenic and anti-inflammatory effects [3]. There is intensive research underway to characterize nutritional strategies for the therapeutic management of inflammatory syndromes [4]. Dietary supplements prepared from functional proteins derived from foodstuffs [5], from bovine colostrum or milk [6], and from porcine or bovine animal plasma [7,8,9], have all been demonstrated to be effective in the amelioration and even prevention of inflammation in numerous experimental models. Recent evidence also indicates that functional protein supplements can be useful in the clinical management of patients with enteropathy (reviewed by [10]).

Fewer models are available for studies focused on inflammation of the small intestine than for the study of colitis syndromes, probably because the incidence of the former is much lower than that of colonic inflammation syndromes. However, Crohn’s disease usually affects distal regions of the small intestine and some inflammatory pathologies (e.g., those induced by some toxins and xenobiotics) are restricted to the small intestine [11]. We previously characterized a model of mild intestinal inflammation based on the acute administration of the Staphylococcus aureus enterotoxin B (SEB). This toxin causes moderate GALT activation, stimulating cell recruitment and the production of pro-inflammatory cytokines in both organized and diffuse GALT subsystems [7]. The limited magnitude of the GALT response to the SEB challenge makes this model appropriate for the study of dietary approaches that may have modulatory effects on signaling pathways. As a result of immune activation, the expression of junctional proteins, such as zonula occludens-1 and β-catenin, is reduced, which is consistent with the increased in luminal water contents and mucosal permeability [12]. SEB also reduces the expression of mucosal defensins [13] and SGLT1 abundance in villous apex [14].

Spray-dried plasma (SDP) of porcine and bovine origin have been widely used as dietary supplements for farm animals at the time of weaning because they promote growth and reduce both morbidity and mortality [15]. Studies of pigs challenged with pathogenic microorganisms indicate that SDP has anti-inflammatory effects because it reduces the expression of pro-inflammatory cytokines [16]. In our laboratory, we previously identified the lymphoid cell subsets as well as the cytokines and chemokines involved in GALT stimulation that are eventually modulated by plasma protein supplements [7,17]. In all instances, plasma supplements reversed the changes in the ratio of activated-to-regulatory T-lymphocytes that is characteristic of inflammatory syndromes, and promoted the expression of anti-inflammatory cytokines.

Studies of the mechanisms by which SDP mediates anti-inflammatory effects indicate that it lowers the expression of mucosal pro-inflammatory cytokines, reduces activation of Th cells and promotes abundance of the Treg subpopulation. Moreover, the production of anti-inflammatory cytokines (typically interlekin 10 and transforming growth factor β) is also increased and this will counteract the effects of TNF-α and other pro-inflammatory mediators. These changes in the cytokine profile may explain the reduction (or even complete prevention) of the effects resulting from an SEB challenge, namely: increased intestinal permeability, reduced defensin secretion and reduced intestinal glucose uptake capacity.

Inflammatory syndromes are also characterized by cellular proliferation in susceptible tissues because elevated concentrations of pro-inflammatory cytokines increase endothelial permeability and facilitate lymphocyte extravasation [18]. The objective of the present study is to analyze the mechanisms involved in the anti-inflammatory effects of SDP, measuring the expression of signaling and regulatory molecules that enable lymphocyte infiltration into tissues, and the progression and consolidation of the inflammatory syndrome.

## 2. Materials and Methods 

### 2.1. Animals and Diets

Male C57BL/6 mice were supplied by Charles River (Lyon, France) and were kept at the Faculty of Pharmacy’s animal house facility under conditions of constant temperature and humidity, with a 12 h:12 h light/dark cycle and with food and water available ad libitum. Ethical Committee for Animal Experimentation of the University of Barcelona’s and the Catalan government approved the protocols used in this study (registration numbers 565/13 and 7397, respectively).

The animals were weaned at 19 days of age and distributed into experimental groups at random. The groups were: (a) control group: mice fed the control diet; (b) SEB group: mice fed the control diet and challenged with SEB; (c) SEB-SDP group: mice fed the SDP supplemented diet and challenged with SEB. Animals were fed the experimental diets for 14 days and the composition of these diets are detailed in Table 1. On day 32, mice were administered with the *S. aureus* enterotoxin B (SEB; 25 µg i.p./mice; Toxin Technology, Sarasota, FL, USA) or vehicle, and 24 h later, mice were anesthetized by i.p. administration of ketamine (90 mg/Kg; Imalgene^®^, Rhone Mèrieux, Lyon, France) and xilacine (10 mg/kg; Rompun^®^, Bayer, Leverkusen, Germany) followed by cardiac puncture for exsanguination. 

### 2.2. Mesenteric Lymph Node Cell Isolation

The mesenteric lymph nodes were obtained, finely minced and incubated in the solution of digestion composed by RPMI-1640 (Invitrogen, Carlsbad, CA, USA) with 5% inactivated fetal bovine serum, 100,000 U/L penicillin, 100 mg/L streptomycin, 10 mM-HEPES, 2 nM-l-glutamine and 150 U/mL collagenase (Invitrogen, Carlsbad, CA, USA) at 37 °C in a shaker (Thermomixer Comfort Eppendorf^®^, Heuppauge, NY, USA). The mesenteric lymph nodes were mechanically disaggregated and passed through stainless steel mesh. The cell suspension was centrifuged at 500× *g* for 10 min at 4 °C. The pelleted cells were resuspended in PBS-FBS. Cell number and viability were determined using acridine orange and ethidium bromide.

### 2.3. Cell Staining

The procedure was performed as described before [19]. Briefly, the staining was carried out on 1.5 × 10^4^ cells. To stain extracellular markers, cells were incubated with the primary antibodies for 30 min at 4 °C (Table 2). To stain the intracellular markers, cells were fixed with paraformaldehyde and permeabilized with Triton-X^®^ (Sigma, St. Louis, MO, USA). Then, cells were washed and incubated with the primary antibodies to intracellular markers (Table 2). Finally, cells were washed and maintained in paraformaldehyde until further analysis in the Gallios Flow cytometer (Beckman Coulter, Miami, FL, USA), located at the Cytometry Unit of the Cientifico-Technical Services of the Barcelona Science Park. Results were analyzed using the Flowjo Software (version 7.6.5, Treestar Inc., Ashland, OR, USA). Lymphocytes and non-lymphocytic leucocytes were separated by forward/side scatter.

### 2.4. Western Blot Analysis

Western blot procedures were performed according to a previous study [20]. Samples of jejunum mucosa were homogenized and protein concentration was determined using the Bradford method (Bio-Rad, Hercules, CA, USA). Equal amounts of protein (100 µg) were separated on 10% SDS-PAGE and transferred to polyvinylidene difluoride membranes (Bio-Rad, Hercules, CA, USA). Membranes were incubated overnight at 4 °C with diluted primary antibodies against β-actin (clone AC-74; 1:20,000; Sigma, St. Louis, MO, USA), Smad2/3 and its phosphorylated form p-Smad2/3 (clones D7G7 and D27F4, respectively, both 1:750; Cell Signaling Technology, Danvers, MA, USA) and NF-κB and its phosphorylated form p-NF-κB (clones D14E12 and 93H1, respectively; both 1:1000; Cell Signaling Technology, Danvers, MA, USA). Membranes were washed and incubated with horseradish peroxidase (HRP)-conjugated secondary antibodies (Sigma, St. Louis, MO, USA) for 2 h at room temperature. Protein bands were visualized using a chemiluminescence detection kit Clarity and ChemiDoc XRS+ instrument (both from Bio-Rad, Hercules, CA, USA). After their detection, hybridization bands were quantified using ImageJ gel analyzer software [21].

### 2.5. Real-Time PCR Analysis

RNA extraction and reverse transcription were carried out as described previously [17]. RNA quality and quantity were assessed by spectrophotometry (NanoDrop ND-1000; ThermoFisher Scientific, Waltham, MA, USA). Total RNA was retro-transcribed using iScript™ cDNA Synthesis Kit (Bio-Rad, Hercules, CA, USA). For real-time PCR determinations, we used a template of cDNA in a 20 μL reaction containing 0.2 μmol/L of each primer and SsoAdvanced™ Universal SYBR^®^ Green Supermix (Bio-Rad, Hercules, CA, USA). The primers used are listed in the Table 3. Real-time PCR was performed on a MiniOpticon Real-Time PCR System (Bio-Rad, Hercules, CA, USA). Each PCR run included duplicates of reverse transcription for each sample and negative controls (reverse transcription-free samples, RNA-free sample). Quantification of the target gene transcripts was done using hypoxanthine phosphoribosyltransferase 1 (HPRT1) gene expression as reference, and was carried out with the 2^−ΔΔCT^ method [22]. Product fidelity was confirmed by melt-curve analysis.

### 2.6. Statistical Analysis

The data from the experiments are presented as the mean ± SEM. Previous research indicated that 7 to 8 animals are enough to observe SDP effects on immune populations [19], while 4 to 5 samples are sufficient to detect changes in gene expression [17]. Mean values of normally distributed data were compared using one-way ANOVA followed by Fisher least square difference (LSD) post hoc test. Non-normally distributed variables and variables with non-homogeneous variances were log-transformed before analysis. Log-transformed variables were then back-transformed for graphical representation. Statistical tests were performed using Prism (version 6.01, GraphPad Software, Inc., La Jolla, CA, USA). Differences were considered significant at *p* < 0.05.

## 3. Results

### 3.1. Lymphocyte Populations

**Figure 1:**
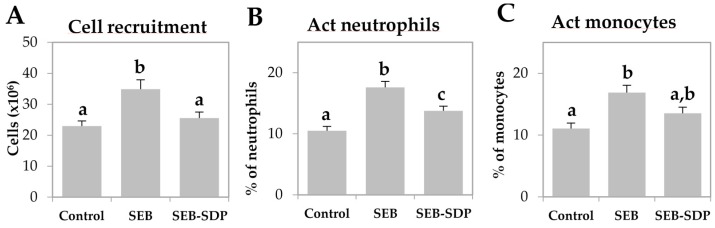

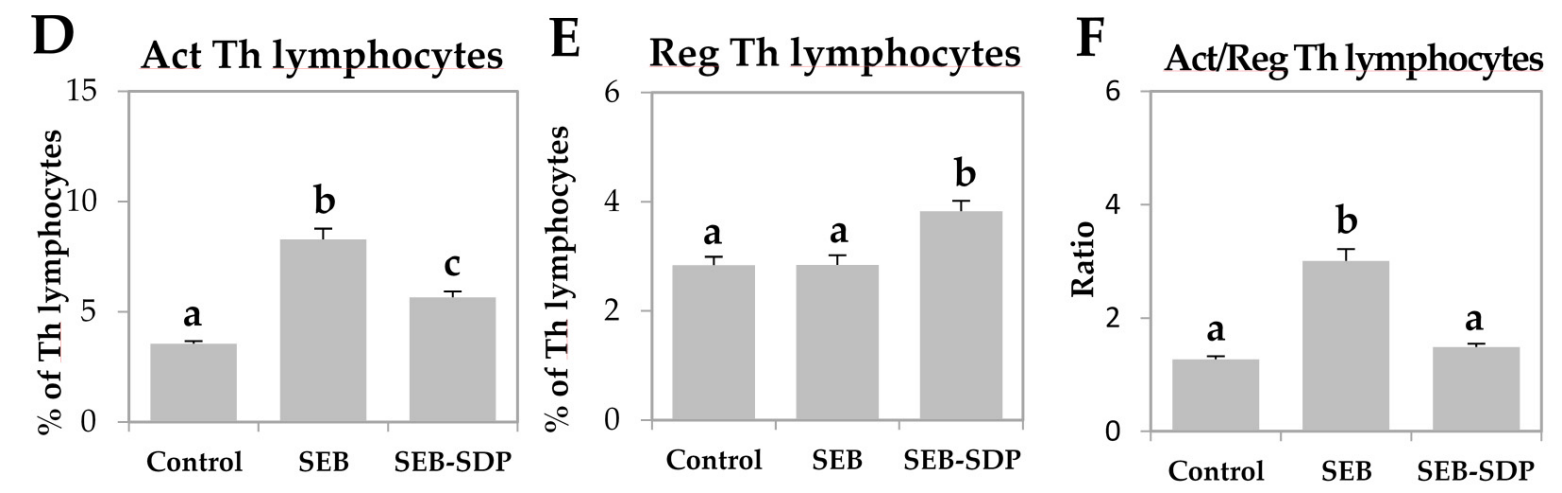
Cell recruitment (**A**); percentage of activated (Act) neutrophils (**B**); Act monocytes (**C**); Act Th lymphocytes (**D**); regulatory (Reg) Th lymphocytes (**E**); and the ratio between Act and Reg Th lymphocytes (**F**) in mesenteric lymph nodes from Control, SEB and SEB-SDP mice. Leukocyte populations were detected by flow cytometry, and CD45^+^ for leucocytes; lymphocytes and non-lymphocytic leucocytes were separated by forward/side scatter; CD68^+^CD14^+^ for Act monocytes, Ly6G^+^CD14^+^ for Act neutrophils, CD4^+^CD25^+^FoxP3^−^ for Act Th lymphocytes and CD4^+^CD25^+^FoxP3^+^ for Reg Th lymphocytes. Groups: Control, reference group; SEB, mice challenged with S. aureus enterotoxin B (SEB); SEB-SDP, mice fed spray-dried plasma (SDP) and challenged with SEB. All of the results are expressed as mean ± SEM (*n* = 7–8 animals). Data were compared by ANOVA followed by Fisher least square difference (LSD) post hoc multiple-comparison tests. Means without a common letter differ, *p* < 0.05.

**Figure 2:**
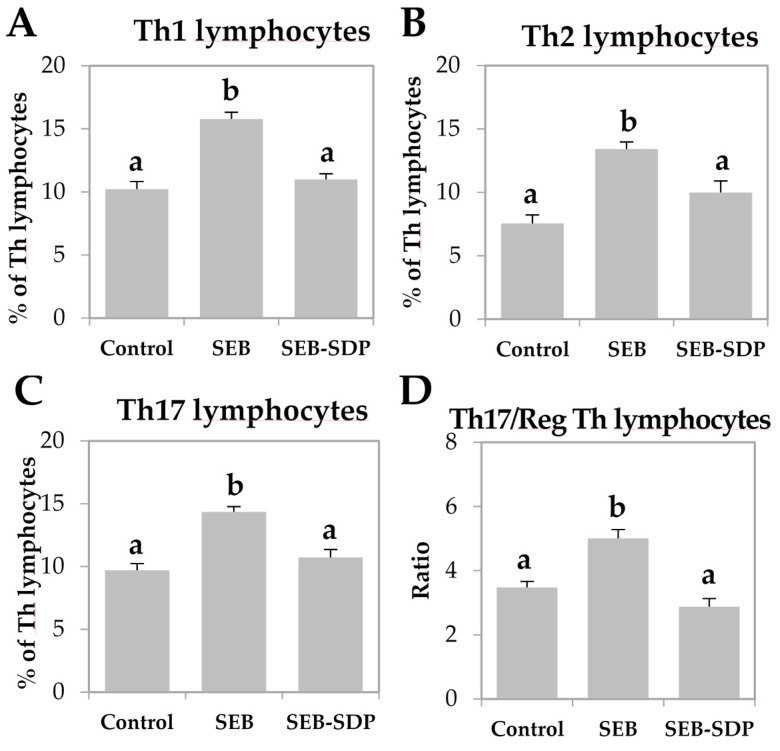
Percentage of Th1 lymphocytes (**A**); Th2 lymphocytes (**B**); Th17 lymphocytes (**C**); and Th17/Treg lymphocytes (**D**) ratio in mesenteric lymph nodes from Control, SEB and SEB-SDP mice. Lymphocyte subsets were detected by flow cytometry, CD4^+^IFN-γ^+^ for Th1 lymphocytes, CD4^+^IL-4^+^ for Th2 lymphocytes and CD4^+^IL-17^+^ for Th17 lymphocytes. IFN-γ, interferon γ. Data were compared by ANOVA followed by Fisher LSD post hoc multiple-comparison tests. All of the results are expressed as mean ± SEM (*n* = 7–8 animals). Means without a common letter differ, *p* < 0.05.

SEB administration induced cell recruitment into mesenteric lymph nodes by 45% (*p* = 0.0291; Figure 1A) and an SDP diet prevented this effect (*p* = 0.0101). Enterotoxin administration increased the percentage of activated neutrophils and activated monocytes (*p* < 0.0001 and *p* = 0.0004; Figure 1B,C, respectively). SDP supplementation completely prevented the SEB effects on the percentage of activated neutrophils (*p* = 0.0032) and partially reduced the percentage of activated monocytes. SEB challenge increased more than two-fold the percentage of activated lymphocytes (*p* < 0.0001; Figure 1D) and had no effect on the percentage of regulatory Th (Treg) lymphocytes (Figure 1E), increasing the ratio between activated and regulatory Th lymphocytes (*p* < 0.0001; Figure 1F). SDP supplementation reduced the effect of SEB on the percentage of activated Th lymphocytes (*p* < 0.0001) and increased the percentage of Treg (*p* = 0.0004), reducing the ratio between activated Th lymphocytes and Treg (*p* < 0.0001). As for Th lymphocytes, enterotoxin administration increased the percentage of Th1, Th2 and Th17 lymphocytes as well as the ratio between Th17/Treg lymphocytes (all *p* < 0.0001; Figure 2) and SDP reduced SEB effects on the percentages of these Th subsets (Th2 lymphocytes, *p* = 0.0017; Th1 and Th17 lymphocytes and Th17/Treg lymphocytes, *p* < 0.0001).

### 3.2. Cytokine Expression

**Figure 3:**
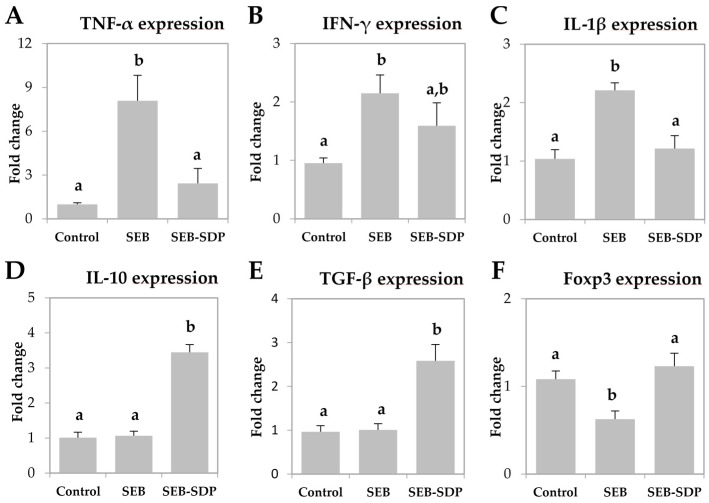
mRNA expression of TNF-α (**A**); IFN-γ (**B**); IL-1β (**C**); IL-10 (**D**); TGF-β (**E**) and Foxp3 (**F**) in jejunum mucosa from Control, SEB and SEB-SDP mice. All target genes were normalized to hypoxanthine phosphoribosyltransferase 1 (HPRT1) expression. TNF- α; tumoral necrosis factor α; IL-1β, interleukin 1 β; IL-10, interleukin 10. Data were compared by ANOVA followed by Fisher LSD post hoc multiple-comparison tests. All the results are expressed as mean ± SEM (*n* = 4–5 animals). Means without a common letter differ, *p* < 0.05.

The enterotoxin increased the expression of pro-inflammatory cytokines TNF-α, IFN-γ and IL-1β in jejunum mucosa (*p* = 0.0020, *p* = 0.0190 and *p* = 0.0023, respectively; Figure 3A–C) and SDP diet attenuated this effect on TNF-α and IL-1β (*p* = 0.0074 and *p* = 0.0057, respectively). SEB administration did not change the expression of IL-10 and TGF-β (Figure 3D,E), but SDP supplementation increased the expression of both cytokines compared to SEB group (*p* = 0.0231 and *p* = 0.0013, respectively). Enterotoxin administration decreased Foxp3 expression in jejunum mucosa and SDP diet attenuated the SEB effect on this variable (*p* = 0.0202 and *p* = 0.0047, respectively; Figure 3F).

### 3.3. Expression of Adhesion Molecules

**Figure 4:**
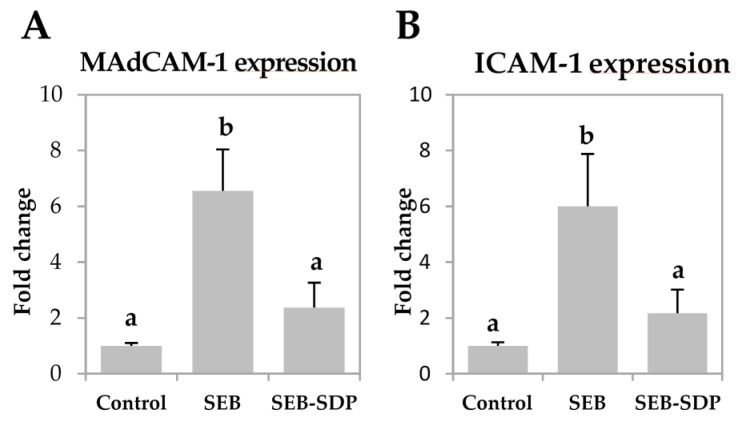

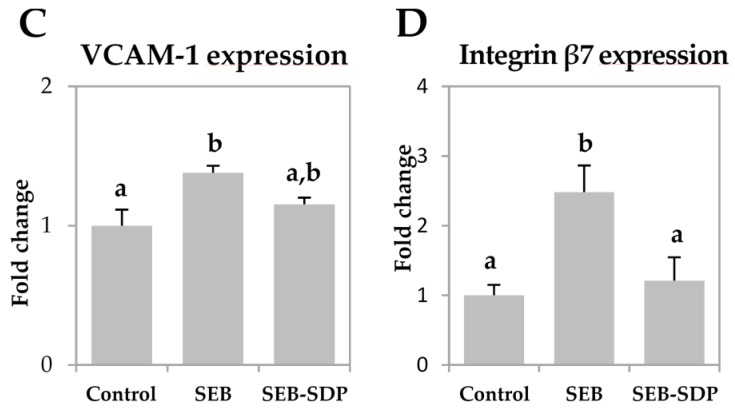
Expression of MAdCAM-1 (**A**); ICAM-1 (**B**); VCAM-1 (**C**) and Integrin β7 (**D**) in jejunum mucosa from Control, SEB and SEB-SDP mice. All target genes were normalized to HPRT1 expression. All the results are expressed as mean ± SEM (*n* = 4–5 animals). MadCAM-1, mucosal addressin cell adhesion molecule 1; ICAM-1, intercellular adhesion molecule 1; VCAM-1, vascular cell adhesion molecule 1. Data were compared by ANOVA followed by Fisher LSD post hoc multiple-comparison tests. Means without a common letter differ, *p* < 0.05.

SEB administration increased the expression of mucosal addressin cell adhesion molecule 1 (MAdCAM-1) and intercellular adhesion molecule 1 (ICAM-1) (*p* = 0.0073 and *p* = 0.0052, respectively; Figure 4A,B) six-fold. It also induced a modest but significant increase in vascular cell adhesion molecule 1 (VCAM-1) expression and a two-fold increase in the expression of integrin β7 in intestinal mucosa (*p* = 0.0190 and *p* = 0.0072, respectively; Figure 4C,D). SDP supplementation prevented an SEB effect on the expression of the adhesion molecules MAdCAM-1, ICAM-1 and integrin β7 (*p* = 0.0198, *p* = 0.0385 and *p* = 0.0217, respectively).

### 3.4. Expression of Transcription Factors

**Figure 5:**
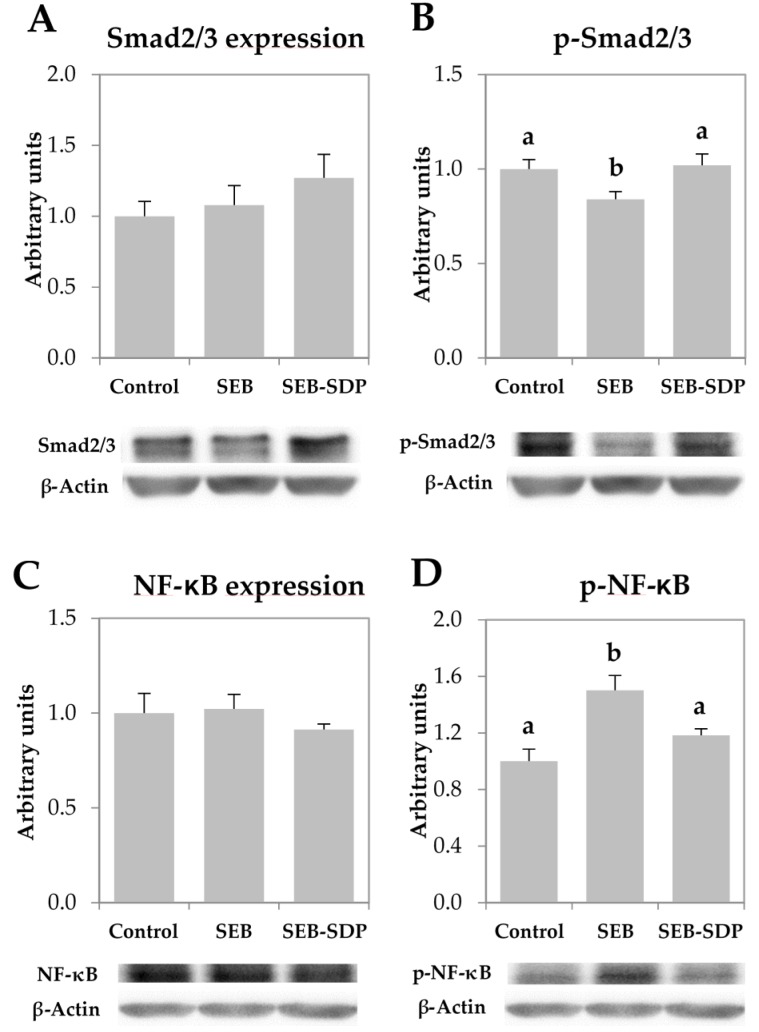
Mucosal amount of Smad2/3 (**A**); p-Smad2/3 (**B**); NF-κB (**C**) p-NF-κB (**D**) in jejunum mucosa from Control, SEB and SEB-SDP mice. All the results are expressed as mean ± SEM (*n* = 4–5 animals). P-Smad2/3, phosphorylated Smad2/3; NF-κB, nuclear factor kappa B; p-NF-κB, phosphorylated nuclear factor kappa B. Data were compared by ANOVA followed by Fisher LSD post hoc multiple-comparison tests. Means without a common letter differ, *p* < 0.05.

The total amount of Smad2/3 and NF-κB was not modified by the enterotoxin nor by SDP supplementation since all groups showed similar value (Figure 5A,C). On the other hand, administration of SEB decreased phosphorylation of Smad2/3 (*p* = 0.0066; Figure 5B) and increased NF-κB phosphorylation (*p* = 0.0010; Figure 5D). SDP supplementation prevented the effect of SEB administration on both transcription factors (Smad2/3 phosphorylation *p* = 0.0016, and NF-κB phosphorylation, *p* = 0.0201).

## 4. Discussion

Dietary supplements prepared from animal plasma are administered to farm animals to minimize the impact of the stress associated with early weaning [5]. Preparations from porcine or bovine plasma can enhance the health and performance of young animals, due in part to improvements in gut barrier function, normalization of cytokine signals, and support of enteric immune function [23]. Several studies have aimed to discover the mechanisms involved in the anti-inflammatory effects of these and other immunoglobulin-rich preparations (e.g., milk-derived proteins). Among other mechanisms [24], their capacity to maintain a homeostatic immune balance in the gastrointestinal mucosa must be part of the explanation. 

We previously demonstrated that SDP reduces the alterations observed in mucosal permeability [25], in epithelial defensin secretion [13], and in glucose uptake capacity [14], which are induced by an SEB challenge. In the present study, we use this well-consolidated model to further explore the effects of SDP on the mucosal expression of cytokines and to analyze whether this supplement can regulate the pathways that signal the infiltration and activation of lymphocytes into the mucosa. 

In mice, the SEB challenge induces intestinal inflammation with features that are similar to those previously described in the rat [12]. In both species, SEB increases infiltration of immune cells into intestinal mucosa and enhances production of pro-inflammatory cytokines, without affecting the histology of the jejunum or systemic (immune) variables. In mice, SEB increased leukocyte recruitment to mesenteric lymph nodes and activation of lymphoid cell populations, thereby confirming previous results [26]. The percentage of T-activated lymphocytes was increased while the T-regulatory subpopulation was not modified, indicating that the change in the Tact/Treg ratio is due to proliferation of the Tact population. The same lymphocyte profile was observed in the colon of mice that spontaneously develop colitis [9].

SEB also increases the percentage of the main Th subsets, namely Th1, Th2 and Th17, consistent with polyclonal stimulation of Th lymphocytes by this enterotoxin [27]. The expression of pro-inflammatory TNF-α and IFN-γ cytokines also increased, as already observed in other models of intestinal inflammation [28]. Increased expression of IL-1β will prime and amplify subsequent intestinal immune responses [29]. This cytokine, acting in concert with other pro-inflammatory cytokines, stimulates Th17 cell differentiation [30] and this, in turn, promotes neutrophil migration to inflammatory sites [31].

SDP supplementation attenuated both innate and acquired immune activation, reducing the percentage of activated neutrophils as well as the relative number of activated Th lymphocytes. Furthermore, SDP reduced the toxin-induced proliferation of Th1, Th2 and Th17 lymphocytes, and also prevented the effects of SEB on the mucosal expression of TNF-α and IL-1β. These effects of SDP are relevant since both TNF-α and IL-1β can induce neutrophilia and neutrophil activation [32]. SDP supplementation also returned the Tact/Treg ratio to control values, confirming previous observations induced by plasma proteins in the inflamed colon mucosa [9] and in the lung and blood of mice challenged with lipopolysaccharide [19]. These effects on the ratio of activated-to-regulatory T-cells are paralleled by changes in the expression of pro- and anti-inflammatory cytokines. Indeed, SDP appears to play a dual role in preventing the SEB-stimulated expression of TNF-α and IL-1β, while increasing 2–3 fold the expression of anti-inflammatory IL-10 and TGF-β cytokines. TGF-β and IL-10 regulate proliferation and differentiation of lymphocytes, phagocytes, dendritic cells and oral tolerance, and thereby modulate the inflammatory response [33]. Another important indicator of the inflammation condition is the Th17/Treg ratio, as it is considered to be critical for host immunity and plays an important role in tolerance preservation and also in autoimmune diseases, including inflammatory bowel diseases [34,35]. 

Tregs are responsible for maintaining immune homeostasis and suppressing intestinal inflammation resulting from aberrant immune responses to self-antigens and commensal bacteria [34]. The expression of signature transcription factor forkhead box p3 (Foxp3) is indispensable for Treg to carry out their anti-inflammatory function [35]. In exploring a possible mechanistic pathway, we found that SDP treatment recovered the otherwise low Foxp3 expression found in SEB-challenged mice. Similar effects of plasma protein supplements were observed in the colitis mouse model [9] and in the acute lung inflammation model [8]. 

A key step in the progression of inflammation is lymphocyte migration into tissues. The SEB challenge stimulates the release of inflammatory mediators and the recruitment of circulating leukocytes, which become activated at the site of inflammation and then stimulate a further release of inflammatory mediators. T cells primed in Peyer’s patches and mesenteric lymph nodes preferentially express α4β7-integrin, while endothelial cells of venules present in the intestinal mucosa constitutively express MAdCAM-1, which is a ligand for α4β7-integrin. This will allow newly induced lymphoid tissue to enter its mucosal effector sites [36].

TNF-α induces the expression of MAdCAM-1 in the small and large intestine, and in mesenteric lymph nodes [37], via a mechanism that requires tyrosine kinases, p38 mitogen-activated protein kinases, and NF-κB [38]. TNF-α and other pro-inflammatory cytokines also stimulate the expression of adhesion molecule receptors and ligands such as VCAM-1 and ICAM-1, which will result in a sustained influx of inflammatory cells [39]. VCAM-1 expression in the gut microvasculature is directly proportional to leukocyte infiltration and histological damage [40] and is regulated by the PI3K/NF-κB signaling pathway [41]. NF-κB activation also plays a predominant role in the induction of ICAM-1 gene expression by TNF-α and thrombin in endothelial cells [42]. 

SDP supplementation reduced the expression of TNF-α and IL-1β, as well as the phosphorylation of NF-κB, suggesting a possible mechanistic pathway of SDP immune modulation, since immunoneutralization of MAdCAM-1, or its leukocyte counter-receptor integrin α4β7, has been shown to result in significant clinical benefits in experimental and human inflammatory bowel disease [43,44]. Reduction in ICAM-1 expression would explain previous results using the mouse colitis model in which plasma protein supplementation reduced leukocyte infiltration into the colon mucosa [9,17]. Other supplements with anti-inflammatory properties, such as ginkgo leaf extracts [45], or drugs such as the β-adrenergic antagonist carvedilol [46] can also inhibit endothelial adhesion to monocytes by reducing VCAM-1 expression via suppression of NF-κB signaling pathways.

TGF-β regulates tight-junction proteins and the permeability properties of the mucosa, which determines the severity of the inflammatory syndrome [47]. The observation that SDP supplementation increases the expression of TGF-β could explain the improvement in barrier function observed in rats challenged with SEB [24] and in mice that develop colitis [9,17]. The role of TGF-β in barrier function is partially mediated by Smad2/3 signaling pathways [47]. Our results show that SEB reduces the phosphorylated form of Smad2/3 and SDP reversed this effect. Consequently, we can tentatively conclude that the protective effect of SDP on the intestinal epithelial barrier observed in different experimental models of intestinal inflammation involves TGF-β canonical Smad pathways. Xiao et al. [48] also show, in a model of intestinal inflammation induced by lipopolysaccharide, that a whey protein concentrate protects intestinal integrity via a mechanism that also involves TGF-β and phosphorylation of the Smad2/3 complex pathway.

SDP is thus shown to have anti-inflammatory effects by reducing the expression of pro-inflammatory cytokines and both the proliferation and expansion of lymphocyte populations in intestinal and peripheral tissues. Although the initial steps that connect SDP components with GALT populations are still not completely understood, they probably involve bioactive peptides present in plasma, a reduction of the luminal antigen charge and promotion of a beneficial microbiota profile. Whatever the trigger is, what we now know is that SDP prevents lymphocyte migration into tissues while reducing the expression of adhesion molecules via mechanisms involving phosphorylation of specific signaling pathways. Moreover, SDP can also reduce NF-κB phosphorylation, which is a key regulator of important inflammatory cascades.

## 5. Conclusions

Our findings indicate that the anti-inflammatory effects of SDP involve a reduction in the expression of adhesion molecules which decreases mucosal cell infiltration. Moreover, SDP can reduce NF-κB phosphorylation, resulting in a reduction in leukocyte activation. These results support the view that oral supplements rich in immunoglobulins and bioactive peptides may play a role in restoring mucosal homeostasis when challenged by pro-inflammatory antigens and pathogens.

## Figures and Tables

**Table 1:** Composition of experimental diets.

Ingredients	Control Diet (g/kg)	SDP ^1^ Diet (g/kg)
SDP	-	80.0
Corn starch	199.3	272.5
Dried skim milk	530.7	376.4
Sugar	100.0	100.0
Soybean oil	70.0	70.7
Cellulose	50.0	50.0
AIN-93 VX ^2^	10.0	10.0
AIN-93 MX ^2^	35.0	35.0
dl-Methionine	2.5	2.9
Choline bitartrate	2.5	2.5

^1^ SDP (Spray-dried plasma) is provided by APC-Europe, Granollers, Spain; ^2^ AIN-93 VX, Vitamin mix; AIN-93 MX, mineral mix. Both were provided by Harlan Ibérica, Barcelona, Spain.

**Table 2:** Antibodies used for cell staining.

Antigen	Cell Subtype	Clone	Source
CD45	present in all leukocytes	30-F11	eBioscience
CD4	Th cells	RM4-5	eBioscience
CD25	activated T lymphocytes	PC61.5	eBioscience
Foxp3	regulatory T lymphocytes	FJK-16s	eBioscience
CD68	monocytes	FA-11	Bio-Rad
Ly6G	neutrophils	1A8	Becton Dickinson
CD14	activated neutrophils/monocytes	Sa2-8	eBioscience
IL-4	Th2 lymphocytes	11B11	eBioscience
IFN-γ	Th1 lymphocytes	XMG1.2	eBioscience
IL-17	Th17 lymphocytes	EbioTC11-18H10.1	eBioscience

Foxp3, forkhead box P3; IFN- γ, interferon γ; IL-4, interleukin 4; IL-17, interleukin 17; Th lymphocytes, T helper lymphocytes. Becton Dickinson (Franklin Lake, NJ, USA); eBioscience (San Diego, CA, USA); Bio-Rad (Hercules, CA, USA).

**Table 3:** Primers used for real time PCR.

Primer	Forward (5′–3′)	Reverse (5′–3′)	Fragment Size	Accession Number
Foxp3	TTCCTTCCCAGAGTTCTTCCAC	ATGGCCCATCGGATAAGGGT	93 bp	NM_001199347.1
HPRT1	TGGATACAGGCCAGACTTTGTT	CAGATTCAACTTGCGCTCATC	163 bp	NM_013556.2
ICAM-1	CAGTCGCTGTGCTTTGAGAA	GAGGTCTCAGCTCCACACTCT	98 bp	NM_010493.2
IFN-γ	CCTTCTTCAGCAACAGCAAGGCG	CTTGGCGCTGGACCTGTGGG	87 bp	NM_008337.3
IL-1β	TGTGAAATGCCACCTTTTGA	GGTCAAAGGTTTGGAAGCAG	94 bp	NM_008361.4
IL-10	GGCGCTGTCATCGATTTCTCCCC	TGGCCTTGTAGACACCTTGGTCTT	102 bp	NM_010548.2
Integrin β7	GTATCAGGAGCTGAGACAGTTGATT	AGTCACAGTGGATGACAGGC	118 bp	NM_013566.2
MAdCAM-1	CCCATGGCCACAGCTACCTCA	CCCTGGCCCTAGTACCCTAC	85 bp	NM_013591.2
TGF-β	CAGTGGCTGAACCAAGGAGACGG	CCCCGACGTTTGGGGCTGATC	119 bp	NM_011577.1
TNF-α	CCACCACGCTCTTCTGTCTAC	AGGGTCTGGGCCATAGAACT	103 bp	NM_013693.2
VCAM-1	CCTCGCTAGGTTACACAGTGG	TGGGGGCAACGTTGACATA	86 bp	NM_011693.3

Foxp3, forkhead box P3; HPRT1, hypoxanthine phosphoribosyltransferase 1; ICAM-1, intercellular adhesion molecule 1; IFN- γ, interferon γ; Interleukin 1 β, IL-1 β; Interleukin 10, IL-10; MadCAM-1, mucosal addressin cell adhesion molecule1; TGF- β, transforming growth factor β; TNF- α, Tumoral necrosis factor α; VCAM-1, vascular cell adhesion protein 1.
